# Supplementary material for: High-Content RNAi Phenotypic Screening Unveils the Involvement of Human Ubiquitin-Related Enzymes in Late Cytokinesis
Source: Cells. 2022 Nov 30;11(23):3862. doi: 10.3390/cells11233862 (PMC9737832; doi:10.3390/cells11233862)
Supplement: Supplementary file 1 [file cells-11-03862-s001.zip › Supplementary Figure S1.pdf]

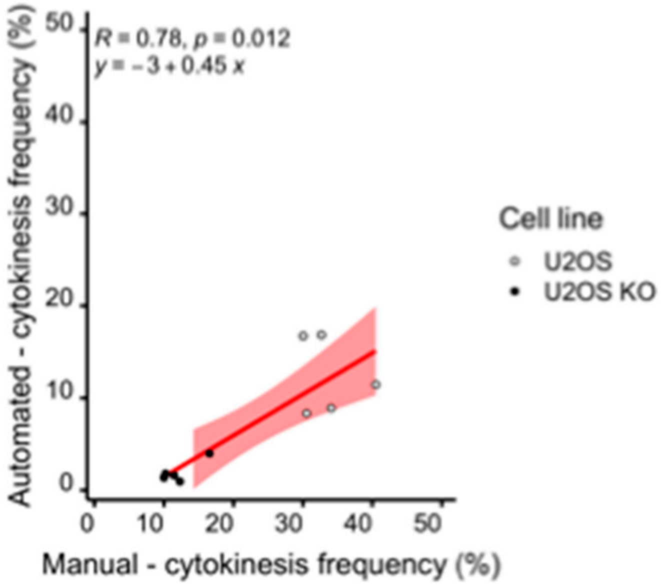

Supplementary Figure S1. Performance of the bioimage analysis tool in WT and *CEP55* KO U2OS cells. The frequencies of cytokinetic bridges from a blind manual detection of intercellular bridges were plotted against those obtained automatically by the program. The dots represent 10 experimental conditions – either WT or CEP55 KO cells – from five different experiments. The correlation between the results of the two methods was appreciated by a Spearman correlation test.
