# Supplementary material for: High-Content RNAi Phenotypic Screening Unveils the Involvement of Human Ubiquitin-Related Enzymes in Late Cytokinesis
Source: Cells. 2022 Nov 30;11(23):3862. doi: 10.3390/cells11233862 (PMC9737832; doi:10.3390/cells11233862)
Supplement: Supplementary file 1 [file cells-11-03862-s001.zip › Supplementary Figure S3.pdf]

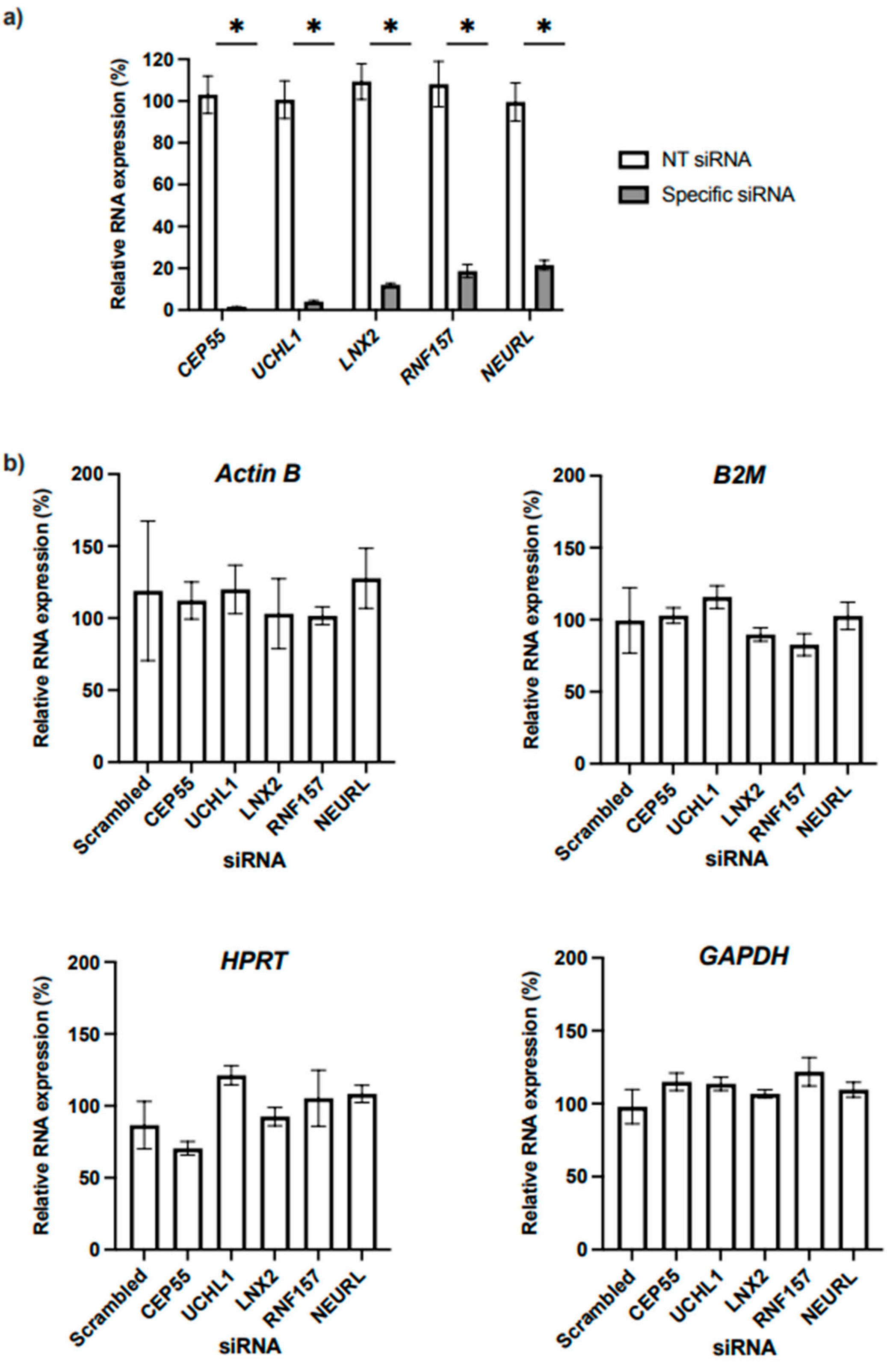

Supplementary Figure S3. Specific siRNA knock-down of *cep55* and candidate genes. HeLa cells were transfected with 25 nM non-targeting scrambled or gene-specific siRNA. 48 hours after transfection, total RNA was extracted, and gene expression was measured by RT-qPCR. The number of transcripts was normalised to the expression of the housekeeping gene *ppia*. (a) The relative expression of *cep55*, *uchl1*, *lnx2*, *rnf157* and *neurl* shows the significance of the respective downregulation. (b) As an indication of specificity, none of the other assessed genes were downregulated after transfection with a *cep55*- or candidate gene-specific siRNA. Shown is the mean +/- SD from four replicates per condition. Wilcoxon-Mann-Whitney tests compare the distributions of the replicates, where the scrambled siRNA condition is the reference. * $p < 0.05$.
