# Supplementary figures and images for: High-Content RNAi Phenotypic Screening Unveils the Involvement of Human Ubiquitin-Related Enzymes in Late Cytokinesis
Source: Cells. 2022 Nov 30;11(23):3862. doi: 10.3390/cells11233862 (PMC9737832; doi:10.3390/cells11233862)

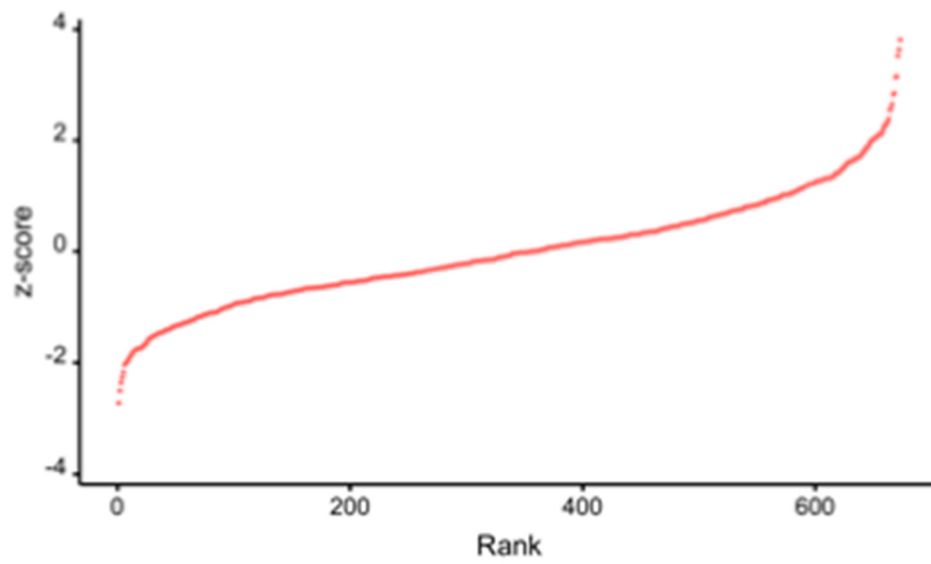

Supplementary Figure S2. Z-score distribution of the screening results ranked by z-score.

Supplement: Supplementary file 1 [file cells-11-03862-s001.zip › Supplementary Figure S2.pdf]
